# Association between prenatal air pollution exposure and risk of hypospadias in offspring: a systematic review and meta-analysis of observational studies

**DOI:** 10.18632/aging.202698

**Published:** 2021-03-19

**Authors:** Ze Xing, Shuang Zhang, Yu-Ting Jiang, Xiu-Xia Wang, Hong Cui

**Affiliations:** 1 Center of Reproductive Medicine, Shengjing Hospital of China Medical University, Shenyang, China; 2 Department of Clinical Epidemiology, Shengjing Hospital of China Medical University, Shenyang, China; 3 Clinical Research Center, Shengjing Hospital of China Medical University, Shenyang, China; 4 Department of Obstetrics and Gynecology, Shengjing Hospital of China Medical University, Shenyang, China

**Keywords:** air pollution, hypospadias, observational studies, risk, meta-analysis

## Abstract

Background: The findings of associations between prenatal air pollution exposure and hypospadias risk in offspring are inconsistent. No systematic review or meta-analysis has yet summarized the present knowledge on the aforementioned topic.

Methods: Relevant manuscripts were identified by searching PubMed and Web of Science databases through January 31, 2020. Summary odds ratios (ORs) with 95% confidence intervals (CIs) in meta-analyses were estimated based on a random effects model. Publication bias was evaluated by funnel plots, Begg’s test, and Egger’s test.

Results: The search identified 3,032 relevant studies. Sixteen studies cumulatively involving 21,701 hypospadias cases and 1,465,364 participants were included. All of these studies were classified as having a low risk of bias. We classified pollutants as nitrogen oxides, particulate matter (PM), ozone, and other exposures. The exposure window to pollutants varied from three months before conception to seven days after delivery. In the meta-analyses, only PM_2.5_ exposure in the first trimester was related to increased risk of hypospadias (per 10 μg/m^3^ OR = 1.34; 95% CI: 1.06–1.68).

Conclusion: We found evidence for an effect of PM_2.5_ exposure on hypospadias risk. Improvements in the areas of study design, exposure assessment, and specific exposure window are needed to advance this field.

## INTRODUCTION

Air pollution has become a major public health concern during the past decade [1]. Increasing evidence is suggesting that common pollutants, such as nitrogen oxide (NO_2_), sulfur dioxide (SO_2_), particulate matter (PM), and carbon monoxide (CO), are connected with a number of adverse health events, including chronic obstructive pulmonary diseases [2], lung cancer [3], and cardiovascular diseases [4]. Notably, increasing epidemiologic evidence also shows that prenatal exposure to ambient air pollution could affect several pregnancy and fetus outcomes [5–8]. Among these outcomes, congenital anomalies are one of the leading causes of perinatal death, accounting for 10% of deaths worldwide in children younger than 5 years of age [9, 10]. Previous studies found prenatal air pollution exposure might result in the development of several congenital abnormalities [11–14].

Hypospadias, which occurs between 8 to 16 weeks of gestation [15], is one of the most common congenital disorders of the male urogenital system. Hypospadias is a complex congenital disease originating from a variety of interacting genetic and environmental aspects [16]. Previous observational studies have yielded controversial conclusions regarding the relationship between prenatal exposure to air pollution and the risk of hypospadias. Some studies found that prenatal exposure to PM_2.5_ and heavy metal hazardous air pollutants increased hypospadias risk [10, 17–21]. However, some studies have reported that urogenital anomalies are not associated with exposure to NO_2_, PM_10_, PM_2.5_, and heavy metal hazardous air pollutants (HMHAPs) in early pregnancy [19–22]. The conflict might be attributed to small sample size, regional environmental air pollution, diverse assessments and windows of prenatal exposure, and adjustments for confounders. Although several meta-analyses have evaluated the link between air pollution and several birth defects [12–14], mainly in cardiovascular malformation and cleft lip and palate, none of them focused on hypospadias. As far as we know, there has been no systematic review to evaluate prenatal exposure to air pollution with hypospadias risk. Therefore, to summarize the evidence of the aforementioned topic, we conducted the present systematic review and meta-analysis based on the latest observational researches.

## RESULTS

### Study selection

**Figure 1:**
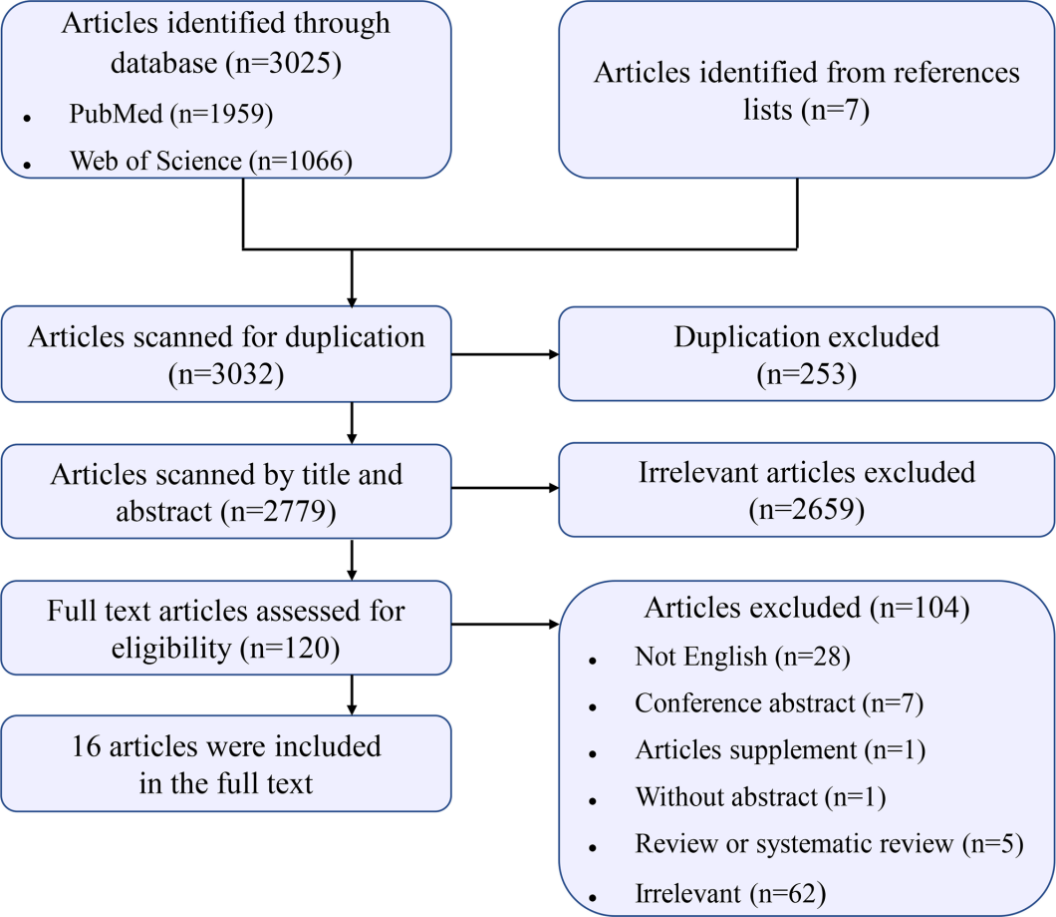
**Flow chart for study selection.**

The detailed process of study selection is illustrated in Figure 1. Briefly, there were 3,032 records identified by searching the databases and reference lists. After screening by title, we removed 253 records for duplication. Subsequently, 2,779 records were assessed by reviewing the titles and abstracts. In 120 records reviewed in the full text, we finally included 16 records that investigated an association between prenatal exposure to air pollution and hypospadias risk [10, 17–31].

### Characteristics and quality assessment of the included studies

The key characteristics of the included studies are reported in Supplementary Table 1. Sixteen studies (three cohort and thirteen case-control studies) were published between 1998 and 2020, with 21,701 hypospadias cases and 1,465,364 participants. Eight studies were conducted in Europe [10, 17, 22–26, 29], six in North America [18, 19, 21, 27, 28, 30], and two in Asia [20, 31]. The exposure windows ranged from 3 months before conception to seven days after delivery. The exposure to various pollutants was assessed mainly using model methods, including the Poisson regression model, hierarchical Bayesian model, spatial land-use regression model, the California Line Source Dispersion Model, and the 2005 NATA Hazardous Air Pollutant Exposure Model. Based on the Evidence-Based Medicine tool, the majority of included studies were level III and only two cohort studies had higher levels of evidence (level II). Detailed findings of meta-analysis on the correlation between prenatal air pollution and hypospadias risk are shown in Supplementary Table 2. In the preliminary analysis of the included studies, some covariates were adjusted (Table 1). The mean study quality score of the included studies was 8.63 (SD = 0.50) out of 9 on the NOS, representing that all these included studies were of adequately high quality (Tables 2, 3).

**Table 1:** Covariates adjusted in primary analyses of included studies.

**First author [Ref]**	**Covariates adjustment in primary study analysis**
Dolk et al. [23]	Socioeconomic status and maternal age
Elliott et al. [24]	Deprivation, year, region
Morris et al. [25]	Year of birth, sex (birth weight and stillbirth only) and deprivation
Cordier et al. [26]	Maternal age, year of birth, and department of birth, population density, family income, and the supplementary information about road traffic
Padula et al. [27]	Maternal race/ethnicity, education, and vitamin use
Vinikoor-Imler et al. [28]	Maternal race (indicator), maternal age, and rural-urban continuum codes category, maternal education, parity, maternal smoking during pregnancy, marital status, prenatal care began in first trimester, and season of birth
Schembari et al. [17]	Maternal age, conception season, year of birth/termination, socioeconomic index
Vinikoor-Imler et al. [18]	Prenatal care in the first trimester, number of previous live births, maternal age, maternal educational attainment, and maternal race/ethnicity
Landau et al. [31]	Individual factors and household environment
Vinceti et al. [29]	Average exposure to the other pollutant in conditional logistic regression, except for reduction deformities of the limb, syndactyly, polydactyly, anomalies of abdominal wall and hypospadias, maternal age
Ren et al. [19]	Maternal age, race/ethnicity, pre-pregnancy diabetes, smoking status, marital status, educational level, season of conception, infant sex
Salavati et al. [10]	Maternal age, sex of child, level of education, season of conception, smoking, folic acid use and area-level socioeconomic -score
Sheth et al. [30]	Birth year, race/ethnicity, poverty
White et al. [21]	Maternal race/ethnicity and birth year
Parkes et al. [22]	Maternal age, year of birth, sex, multiple birth, area level ethnicity, deprivation, other sources of emissions, Incinerator road density, subject road density, smoking proxy, individual ethnicity
Huang et al. [20]	Maternal age, birth weight, season of conception, annual household income and population density of residential township, maternal diabetes and hypertension, maternal smoking, and birth year

**Table 2:** Quality assessment of included cohort studies on the basis of the Newcastle-Ottawa Scale (NOS).

**First author [Ref], year**	**Selection**				**Comparability**	**Outcome**			**NOS score**
	**Representativeness of the exposed cohort**	**Selection of the unexposed cohort**	**Ascertainment of exposure**	**Outcome of interest not present at start of study**	**Control for important factor or additional factor^a^**	**Assessment of outcome**	**Follow-up long enough for outcomes to occur**^b^	**Adequacy of follow-up of cohorts**^c^	
Landau et al. [31], 2015	*	*	*	*	*	*	*	*	8
Ren et al. [19], 2018	*	*	*	*	**	*	*	*	9
Parkes et al. [22], 2020	*	*	*	*	**	*	*	*	9

A study could be awarded a maximum of one point for each item except for the item Control for important factor or additional factor.

^a^A maximum of 2 points could be awarded for this item. Studies that controlled for maternal age received one point, whereas studies that controlled for other important confounders such as reproductive factors, other pollutions received an additional point.

^b^A cohort study with a median follow-up time ≥16 weeks was assigned one point.

^c^A cohort study with a follow-up rate >75% was assigned one point.

**Table 3:** Quality assessment of included case-control studies on the basis of the Newcastle-Ottawa Scale (NOS).

**First author [Ref], year**	**Selection**				**Comparability**	**Exposure**			**NOS score**
	**Adequate definition of cases**	**Representativeness of cases**	**Selection of control subjects**^a^	**Definition of control subjects**	**Control for important factor or additional factor**^b^	**Exposure assessment**	**Same method of ascertainment for all subjects**	**Non- Response rate**^c^	
Dolk et al. [23], 1998	*	*	*	*	**	*	*	*	9
Elliott et al. [24], 2001	*	*	*	*	*	*	*	*	8
Morris et al. [25], 2003	*	*	*	*	*	*	*	*	8
Cordier et al. [26], 2004	*	*	*	*	**	*	*	*	9
Padula et al. [27], 2013	*	*	*	*	*	*	*	*	8
Vinikoor-Imler et al. [28], 2013	*	*	*	*	**	*	*	*	9
Schembari et al. [17], 2014	*	*	*	*	**	*	*	*	9
Vinikoor-Imler et al. [18], 2015	*	*	*	*	**	*	*	*	9
Vinceti et al. [29], 2016	*	*	*	*	**	*	*	*	9
Salavati et al. [10], 2018	*	*	*	*	**	*	*	*	9
Sheth et al. [30], 2019	*	*	*	*	*	*	*	*	8
White et al. [21], 2019	*	*	*	*	*	*	*	*	8
Huang et al. [20], 2020	*	*	*	*	**	*	*	*	9

A study could be awarded a maximum of one point for each item except for the item Control for important factor or additional factor.

^a^One point was assigned if the control subjects were population-based.

^b^A maximum of 2 points could be awarded for this item. Studies that controlled for maternal age received one point, whereas studies that controlled for other important confounders such as reproductive factors, other pollutions received an additional point.

^c^One point was assigned if there was no significant difference in the response rate between control subjects and cases by using the chi-square test (P>0.05).

### Risk of bias evaluation

**Figure 2:**
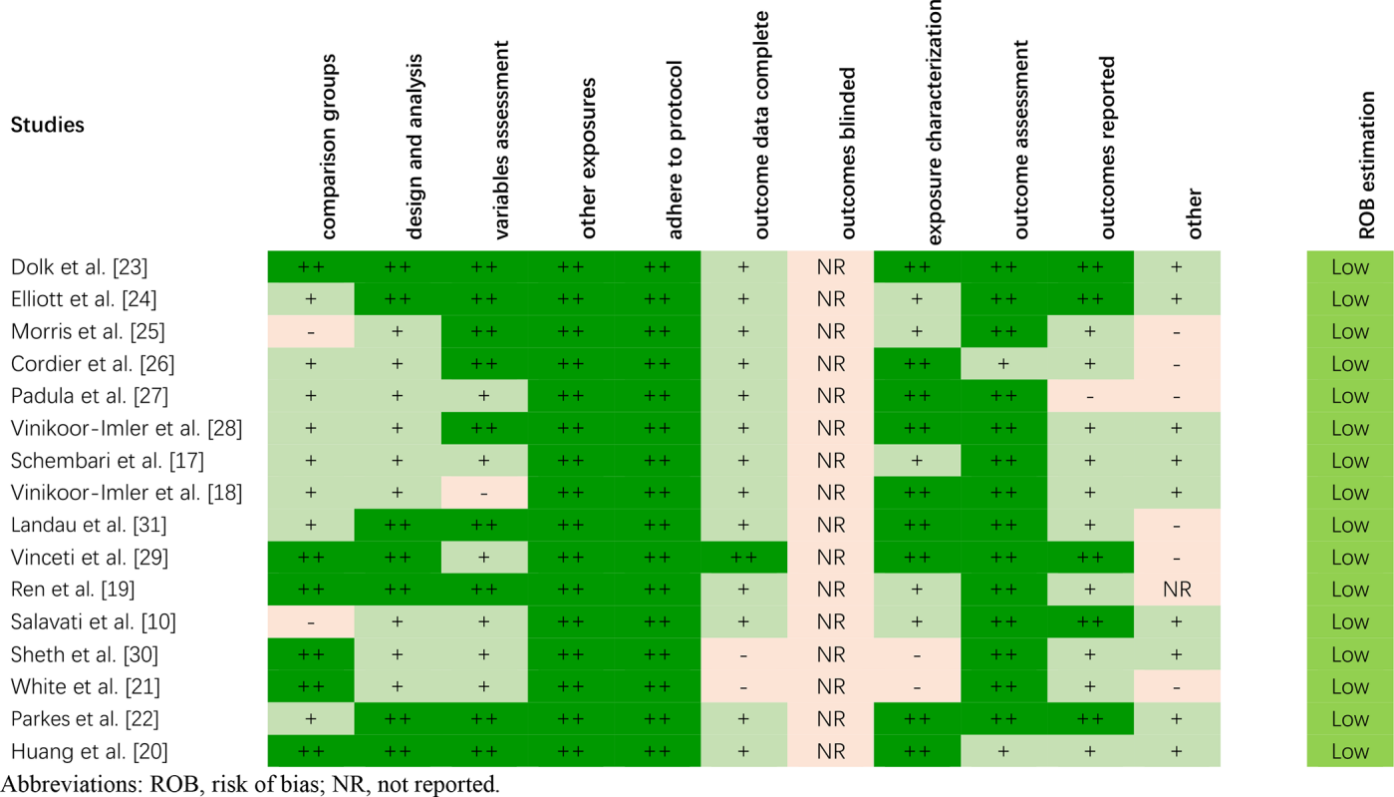
**The risk of bias of included studies on the basis of the OHAT.**

The risk of bias of included studies was evaluated according to OHAT criteria. Overall, low risk was assessed for most of the studies in this systematic review, especially for the selection bias, confounding bias, and performance bias (Figure 2). It was not amazing because nearly all the studies included used analogical study designs (i.e., cohort and case control studies). For most of the studies evaluated, the risk of bias for confounding and exposure misclassification categories was “low” or “possibly low”, mainly because the study design focused on daily variations in air pollution and the available health consequences.

Due to the lack of basic information provided for the control group, two studies [10, 25] were assessed as “possibly high” risk for selection bias (comparison group). Two studies [21, 30] received a “probably high” risk of bias for attrition/exclusion bias (outcome data complete) because the absence of some important data/populations affected the evaluation of the subsequent results. Two studies [21, 30] were also rated as “probably high” risk in terms of detection bias (exposure characterization) due to more than 25 percent of data missing. None of the studies included reported whether the outcome assessors were blinded to the study group and exposure level. One study [27] received a “probably high” risk of bias for selective reporting bias (outcomes reported), as 26 birth defects were mentioned in the method but only 11 were reported in the result. The other 15 birth defects were not presented because the cases were less than 40.

In general, we observed consistent results across the risk assessments for bias in different studies. The analogy in study design explains the consistency of the bias rating risks allocated across the studies. Our literature search revealed 16 studies considered of sufficient quality to prove the conclusions of our systematic review.

### Prenatal exposure to air pollution and hypospadias risk in offspring Nitrogen oxides (NO_X_) [NO_2_ and nitric oxide (NO)] exposure

Three, five, and one studies were carried out to investigate the relationship between prenatal NO_X_ [10, 17, 20], NO_2_ [10, 17, 20, 27, 31], and NO [27] exposure and hypospadias risk, respectively. Among them, only one cohort study focused on NO_2_ exposure [31]. These studies were conducted in the USA, Spain, the Netherlands, Israel, and China, respectively. The number of cases ranged from 67 to 446, and the number of participants ranged from 443 to 2,634. Four studies focused on the 3 months after conception [17, 20, 27, 31], two studies focused on the 3 months before conception [20, 31], and one study focused on the periconceptional period [10]. In general, prenatal NO_X_, NO_2_, and NO exposures in the 3 months post conception were mainly positively associated with hypospadias though there was no statistical significance. Furthermore, Huang et al. [20] investigated the relationship between NO_X_ as well as NO_2_ exposure and hypospadias risk during the 3 months before pregnancy and other months after pregnancy. No statistically significant results were found. However, Salavati et al. [10] found a statistically significant positive correlation between hypospadias risk and exposure to NO_X_ and NO_2_ during the periconceptional period.

### PM exposure

A total of 10 studies (three cohort and seven case–control studies) investigated the connection between PM exposure and hypospadias risk in seven countries, including the USA, Spain, Italy, the Netherlands, Britain, China, and Israel. Seven, eight, and two studies focused on prenatal exposure to PM_10_ [10, 17, 20, 22, 27, 29, 31], PM_2.5_ [10, 17–20, 27, 28, 31], and PM_2.5–10_ [10, 20], respectively. The number of cases ranged from 3 to 978, and the number of participants ranged from 228 to 711,833. Exposure windows ranged from 3 months before conception to 7 days after delivery. Among these ten studies, eight studies focused on the 3 months after conception. For prenatal PM_10_ and PM_2.5_ exposure during 3 months after pregnancy, three, three, and one studies reported an insignificant positive association [17, 18, 20], insignificant inverse association [27–29], and null association, respectively [22]. Of note, Ren et al. [19] and Huang et al. [20] observed a significant positive association in a limited single month before and after conception. Additionally, Salavati et al. [10] covered a statistically significant positive association between perinatal PM_2.5–10_ exposure and hypospadias risk.

**Figure 3:**
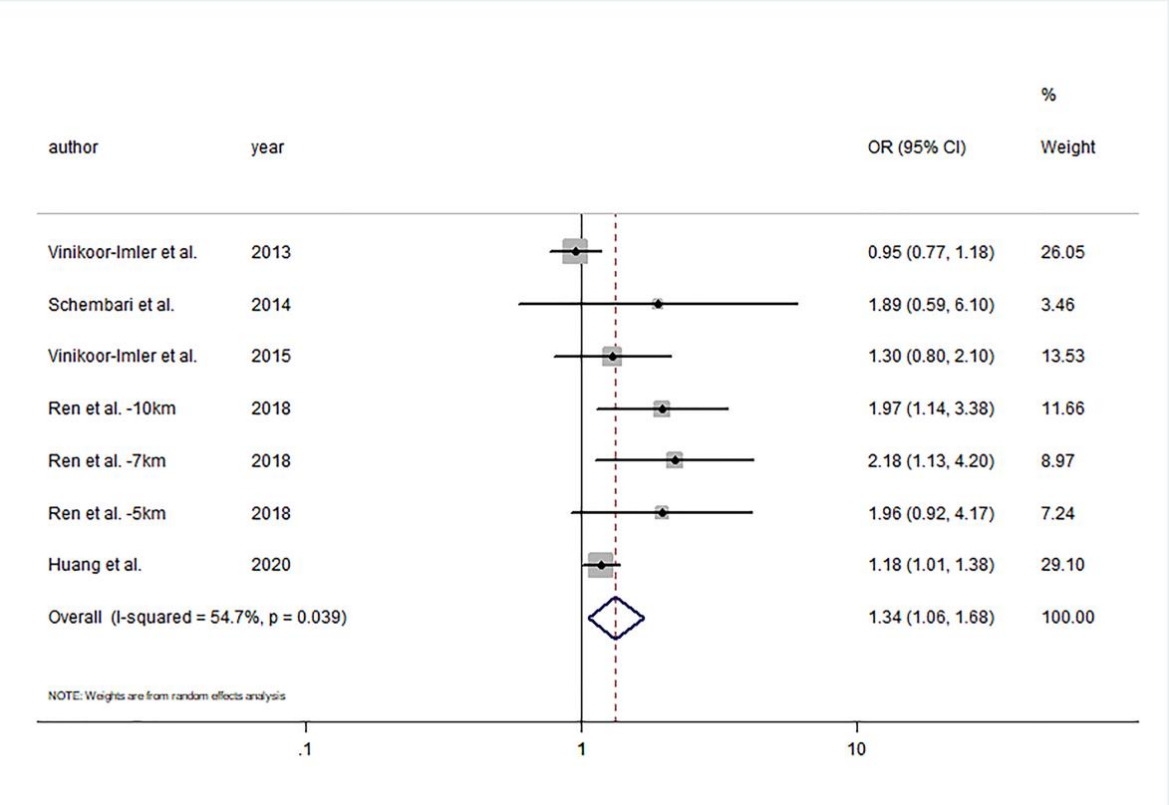
**Forest plot of the association between per 10μg/m^3^ increment in PM_2.5_ in first trimester and risk of hypospadias.**

**Figure 4:**
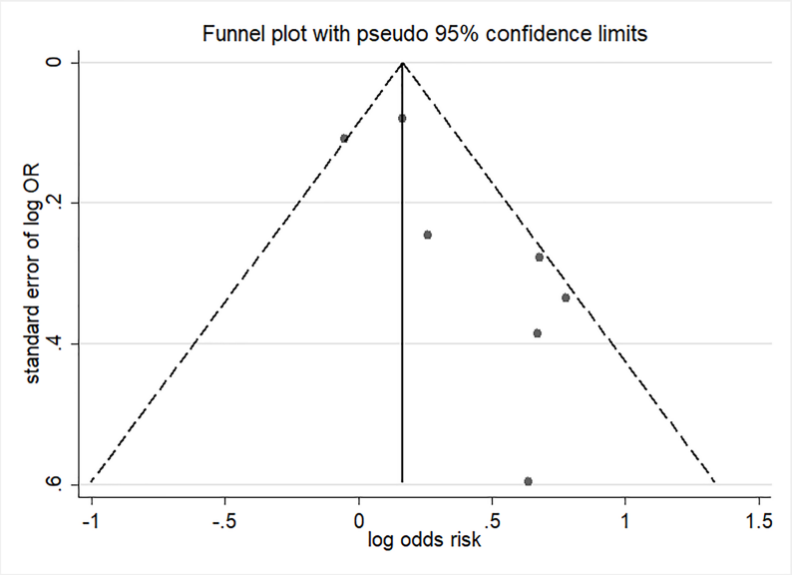
**Funnel plot of per 10μg/m^3^ increment in PM_2.5_ in first trimester and risk of hypospadias.**

Five studies were included in the meta-analysis for the change in hypospadias risk per 10 μg/m^3^ increments in PM_2.5_ by the first trimester period [17–20, 28]. Ren et al. [19] analyzed outcomes using three distance cutoffs including 5, 7, and 10 km from the monitoring station. Exposure to continuous PM_2.5_ during the first trimester was associated with higher odds of hypospadias (OR= 1.34; 95% CI: 1.06, 1.68) with moderate heterogeneity (I^2^ = 54.7%; P = 0.039) (Figure 3). Visual inspection of funnel plot (Figure 4) and the results of Egger’s test (P = 0.06) and Begg’s test (P = 0.37) showed no publication bias.

### O_3_ exposure

**Figure 5:**
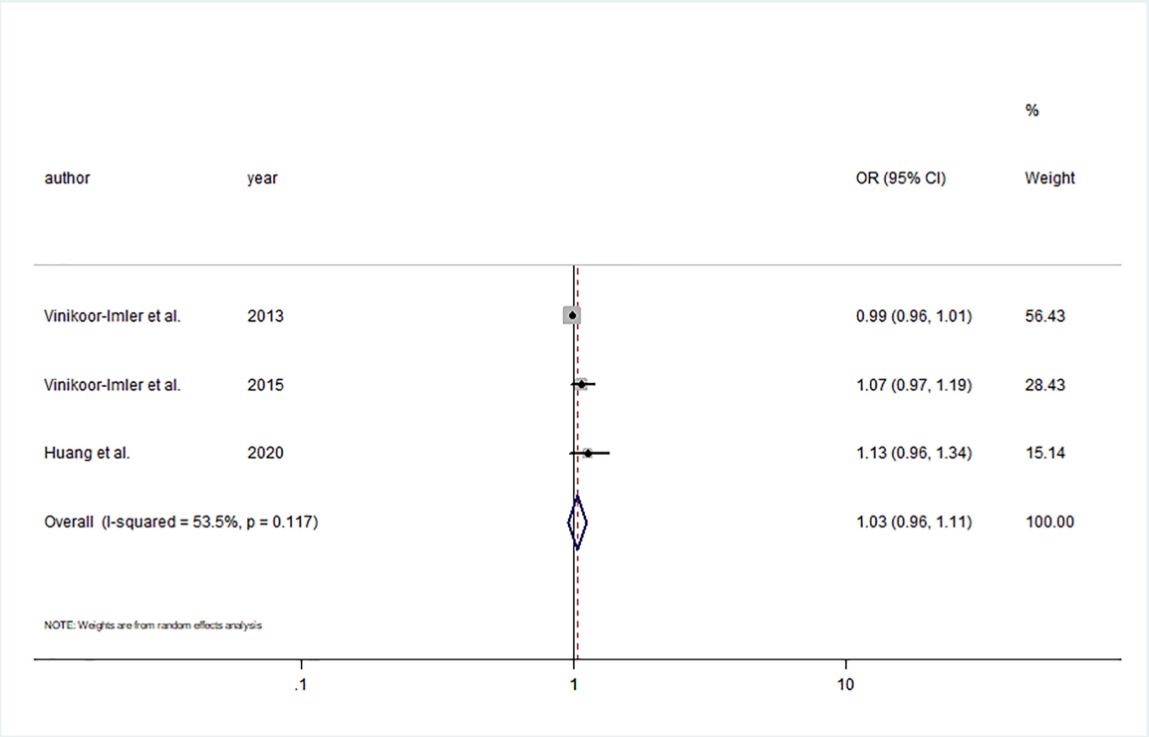
**Forest plot of the association between per 5 ppb increment in O_3_ in first trimester and risk of hypospadias.**

Four case control studies explored the relationship between prenatal O_3_ exposure and hypospadias risk [18, 20, 27, 28]. Three studies were conducted in the USA and one in China. The number of cases ranged from 67 to 978, and the number of participants ranged from 443 to 711,833. Although the exposure windows ranged from 3 months before conception to 6 months after conception, all included studies investigated prenatal exposure in the 3 months after conception. Of these studies, three studies reported an association of continuous O_3_ exposure with the risk of hypospadias [18, 20, 28]. The summary estimate showed that increments of 5 ppb O_3_ during the first trimester were not significantly associated with hypospadias risk (OR= 1.03; 95% CI: 0.96, 1.11; I^2^ = 53.5%) (Figure 5). In addition, significant publication bias was discovered by Egger’s test (P = 0.03).

However, Huang et al. [20] observed significant positive results between O_3_ exposure and hypospadias risk in the first month after conception. Of note, Huang et al. [20] also focused on the aforementioned association not only in the 3 months before conception but also in 6 single months after conception; but, no statistically significant results were found. Two studies [20, 27] investigated the relationship between an 8-hour maximum exposure to O_3_ and hypospadias risk, but no statistically significant result was found.

### Other exposures

Seven case control studies and two cohort studies were included [21–27, 30, 31]. The exposure pollutants were mainly SO_2_, CO, landfill sites, garbage wastes, hormonally active hazardous air pollutants, and HMHAPs in seven countries, including Belgium, Denmark, France, Italy, Britain, the USA, and Israel. Six studies focused on the exposure window in the prenatal period [21, 23–26, 30] and three studies in the 3 months before conception and the first trimester of pregnancy [22, 27, 31]. The number of cases ranged from 45 to 8,981, and the number of participants ranged from 443 to 216,004. Among these studies, Elliott et al. [24] observed a significant positive result between hypospadias risk and exposure in the prenatal period to landfill sites and garbage waste. Padula et al. [27] observed an insignificant positive result between CO exposure and hypospadias risk. In addition, Sheth et al. [30] observed that dimethyl phthalate and pentachlorophenol exposure in the prenatal period had a statistically significant positive association with hypospadias risk. White et al. [21] found a statistically significant positive association between prenatal exposure of HMHAPs and hypospadias risk, except for cadmium and nickel.

## DISCUSSION

As the first systematic review summarizing the association between prenatal exposure to air pollution and hypospadias risk, our study added depth and clarity to the current evidence exploring the aforementioned topic. Meta-analysis suggested that continuous PM_2.5_ exposure during the first trimester was related to statistically significant increases in the risk of hypospadias, but no statistically significant increase in the risk of hypospadias in relation to continuous O_3_ exposure during the first trimester. In the present study, we did not perform further meta-analyses to assess hypospadias with other air pollutants or exposure windows because of the small number of studies presented.

A few established biological processes that occur in the first trimester are related to the development of the urogenital system. Any interference happening in the pivotal window of internal and external urogenital development could become a cause of hypospadias [20]. Recently, there have been several hypothesized biological mechanisms underlying the association between prenatal exposure to air pollution and the risk of birth defects, including hypospadias, such as oxidative stress [32], abnormal coagulation [33], epigenetic changes [34], and placental inflammation [33]. For example, air pollutants, including polycyclic aromatic hydrocarbons and heavy metals involved in PM, especially from diesel exhaust, can act as endocrine disruptors mainly by activating the aryl hydrocarbon receptor or estrogen or androgen receptors [35]. Furthermore, most air pollutants exert their adverse effects by directly acting as prooxidants of lipids and proteins or as free radical generators, promoting oxidative stress and inducing inflammatory responses [36]. Notably, some contaminants can alter DNA molecules or induce epigenetic changes, such as DNA methylation and histone modifications, which can be passed on to offspring [37]. Several studies have suggested that exposure to PM may increase blood viscosity and may interfere with placental functions, which are essential for regulating hormonal climate, particularly in the first trimester of pregnancy [38–40].

Our systematic review had several strengths. Compared to the limited sample size of individual studies, the present review comprehensively included all published observational studies (n = 16), which had relatively large sample sizes. The OHAT risk of bias rating tool was applied to systematically identify underlying risk of bias associated with multiple fields of studies included, while the NOS was used to assess the quality of the studies. Of note, the risk of bias in the included studies was low.

Although we conducted a meta-analysis of some exposures, several challenges should be taken into consideration. First, published studies included in this meta-analysis were too few to carry out a comprehensive analysis. Although studies were each of reasonable quality, high heterogeneity was generated by the different study designs, geographical locations, exposure windows and assessments, and cofounding adjustments. This may have affected the accuracy of the quantifiable findings in the meta-analysis. For instance, Vinceti et al. [29] reported only three cases of hypospadias, which may not represent the true situation of the whole. Therefore, the data available did not allow for a meta-analysis of specific air pollutants, such as PM_10_. Moreover, large well-designed cohort analyses are warranted in the future to explore whether there is a crucial association between air pollution and hypospadias risk.

Second, we could not exclude potential biases due to the misclassification of exposure and outcome. Using a fixed monitor to specify individual-level exposure within a specific radius around the monitor, including land-use regression models, has been widely accepted [19]. However, obvious limitations exist. Based on this method, measurement error of spatial variability might lead to wrong negative consequences, which tends to bias the risk estimation towards the null [19, 41]. Furthermore, air pollution was measured that relied on the location of the birth or maternal residential address, which did not take into account women who moved during their gestation, especially in the early period [10, 19]. Similarly, it did not pay attention to the exposure of non-residential addresses [19]. However, moving behavior would be distributed between the case group and the control group randomly, so the accompanying exposure misclassification might be undifferentiated [10]. On the other hand, outcome ascertainment was different among these included studies. Birth certificate records were commonly used. Nevertheless, compared with the review of medical records, birth certificate records are less sensitive to the identification of birth defects [19]. In addition, some birth defects recorded on the birth certificate may be wrong, thus leading to some misclassification of status in the case or the reference group [19].

Third, the criteria for considering eligible studies for systematic review should be based on a more rigorous PECOS structure. For example, as for exposure, the women in the first trimester of pregnancy were combined as a whole for the meta-analysis because of the small number of articles included. It is well-known that embryonic growth and development occurs in the first trimester of pregnancy, which is the critical time when most teratogenic exposures result in birth defects, including hypospadias. Of note, if the high concentration of certain pollutants or their metabolites accumulate for a long time in the pre-pregnancy period, it may cause a more obvious risk of congenital abnormalities [42]. Interestingly, the included studies focused on different exposure periods. Some studies focused on a short period during the early first trimester only [17, 27, 28]. Some studies focused on the first and second trimesters, while others focused on the entire pregnancy [18, 20–26, 29, 30]. Only four studies investigated the association between preconception exposure to air pollution and hypospadias risk [19, 20, 22, 31]. If specific exposure characteristic studies are sufficient, the meta-analysis of different pregnancy stages could be divided into time periods of one month or even shorter. Ideally, future studies would utilize rigorous exposure window stagings that could be combined to produce aggregated results in future meta-analyses. Caution should be exercised in interpreting the findings.

Fourth, besides the variations in method of measurement and exposure window, the strategies of analyses and forms of results reporting have been different among these studies. Based on the distribution of exposure in each study, air pollution was divided into dichotomy, trisection, and quartering. However, because of the different populations and exposure levels of each study, these aforementioned categories of exposure made it surely complicated to explain and compare the results. Dose-response analysis using a continuous exposure assessment model may provide more informative and nonbiased results, and more accurately reflect the association between ambient air pollution and birth defects than categorical variables, with higher levels of evidence.

Finally, the importance of subgroup data should be recognized and, if possible, future studies should include subgroup analyses of the most relevant socio-demographic and clinical characteristics. Because all individual studies were observational in nature and multivariable analyses were carried out in the primary studies, the chance of residual confounding bias from unscanned variables cannot be excluded. More subgroup analyses stratified by these important potential confounders should be performed in future studies.

In summary, the present meta-analysis indicated that 10 μg/m^3^ increments in PM_2.5_ in the first trimester increased the risk of hypospadias. However, we could not determine the relationship between other ambient air pollutants and hypospadias risk. Further studies that focus on other exposures (e.g., SO_2_), as well as a more accurate assessment of exposures, better case ascertainments, and adjustments for a large number of potentially confounding effects, are needed to provide more evidence toward this topic.

## MATERIALS AND METHODS

This systematic review and meta-analysis were based on the Preferred Reporting Items for Systematic Reviews and Meta-Analyses guidelines [43] and the Meta-Analysis of Observational Studies in Epidemiology guidelines [44].

### Literature search

We implemented a compositive search in PubMed and Web of Science databases to determine all potentially correlative articles from inception through January 31, 2020. The following critical keywords were applied in the literature search: (air pollution) OR (traffic pollution) OR (outdoor pollution) OR (outdoor air pollution) OR (particulate matter) OR (nitrogen dioxide) OR (sulfur dioxide) OR (sulphur dioxide) OR (ozone) OR (carbon monoxide)) AND ((hypospadias) OR (hypospadia) OR (isolated hypospadias) OR (birth defects) OR (congenital anomalies) OR (congenital malformations)). In addition, the bibliography of all included articles and related reviews and meta-analyses were further reviewed to determine whether there are other eligible articles. Searches were limited to English language articles. There is no geographical restriction.

### Selection criteria

Two independent authors (ZX and SZ) deleted duplicates, filtered titles and abstracts for relevance, and determined records as included, excluded, or inconclusive. In case of uncertainty, the two authors obtained the full-text to determine eligibility. Any difference was settled through discussion and negotiation.

Eligible studies were determined on the basis of the following PECOS (population, exposure, comparison/comparator, outcome, and study type) criteria: i) population: our study drew participants from pregnant women and their newborns; ii) exposure: the primary exposure of interest was air pollution on pregnant women; iii) comparison/comparator: the concentration of air pollutants to which the pregnant women were exposed; iv) outcome: the primary outcome of interest was the risk of hypospadias in offspring; the outcomes of other birth defects were not included; and v) study type. We targeted observational study designs (cohort, case–control, nested case–control, or cross-sectional studies); studies without original data (review articles) were not included.

### Data extraction

Data extraction was carried out by SZ and confirmed independently by another author (ZX). The following data were from eligible studies: first author’s family name, publication year, study location, time period, study design, sample size, exposure characteristic (exposure, window, and assessment), and main findings of the results.

### Quality assessment

Two authors (ZX and SZ) independently assessed the quality of the observational studies using the Newcastle-Ottawa Scale (NOS) [45]. The scale consists of 8 items divided into 3 domains: selection of the population (0 to 4 points), comparability of the groups (0 to 2 points), and assessment of the outcome (0 to 3 points). In the selection and outcome categories, each numbered item can earn up to one point. Besides, in the comparability category, up to two points was awarded. Thus, the maximum score for each study was 9 points. The total score reflected the overall quality of the study: scores of 8–9 indicated very good studies, 6–7 indicated good studies, 4–5 indicated satisfactory studies, and 0–3 indicated unsatisfactory studies [46]. The level of evidence was determined using the Oxford Center for Evidence-Based Medicine [47].

### Risk of bias assessment

The Office of Health Assessment and Translation (OHAT) risk of bias rating tool [48] was employed to systematically identify potential risk of bias connected with the multiple domains of each of included studies. Specifically, we examined whether comparison groups were appropriate, whether study design could explain important confounding variables, whether confounding variables were assessed consistently, whether adjustments were made of other exposures, whether researchers followed the study protocol, whether outcome data were complete, whether outcome assessors were blinded to the study group, whether all results were reported, and whether researchers considered other potential threats. We also examined our confidence in the exposure characterization. By establishing a bias risk rating and answering a set of questions founded on the details of the study, two authors (ZX and SZ) independently obtained one of the following judgments: definitely low risk of bias (“++”), probably low risk of bias (“+”), probably high risk of bias (“-” or “NR”: not reported), or definitely high risk of bias (“--”) [49]. Supplementary Table 3 provided extra information on these biases and criteria for assigning a risk of bias category (based on the OHAT risk of bias tool described by Rooney et al. [50], with minor modifications to relevant facets of population-based epidemiologic studies).

### Statistical analysis

If at lowest three studies mentioned the same air pollutant and exposure window of pregnancy available, then the impact estimates were combined in the meta-analyses. We figured summary risk estimates for congenital hypospadias per unit increase of persistent pollutant concentration. If multiple and single pollutant models were reported simultaneously, the single pollutant model analysis was chosen. In addition, if studies demonstrated effect estimate for more than one pregnancy exposure window, we extracted the results during the first trimester of gestation. To compare and contrast among different studies, units were harmonized to per 10 μg/m^3^ increase in PM_2.5_ and per 5 ppb increase in ozone (O_3_). The summary odds ratios (ORs) in the meta-analyses were estimated based on a random effects model, following the method of DerSimonian and Laird [51]. Heterogeneity was quantified using I^2^ statistic. The cutoff points ≤ 25%, ≤50%, ≤75%, and >75% were used to indicate no, small, moderate, and significant heterogeneities, respectively [52]. Furthermore, publication bias was evaluated by funnel plots, Begg’s test, and Egger’s test [53, 54]. STATA version 12.0 (Stata LLC, College Station, TX, USA) software was used for analyses.

## Supplementary Material

Supplementary Table 1

Supplementary Table 2

Supplementary Table 3
