# Supplementary material for: Association between prenatal air pollution exposure and risk of hypospadias in offspring: a systematic review and meta-analysis of observational studies
Source: Aging (Albany NY). 2021 Mar 19;13(6):8865–79. doi: 10.18632/aging.202698 (PMC8034939; doi:10.18632/aging.202698)
Supplement: Supplementary Table 1 [file aging-13-202698-s001.docx]

**Supplementary Table 1. Characteristics of studies included in the meta-analysis for association between prenatal air pollution and risk of hypospadias.**

| **Author [Ref] (year);**  **Study location**  **(time period)** | **Study design/level of evidence** | **Sample size** | **Exposure characteristic** | | | **Main findings (hypospadias/ hypospadias and epispadias)** | | | | |
| --- | --- | --- | --- | --- | --- | --- | --- | --- | --- | --- |
|  |  |  | **Window** | **Assessment** | **Exposure** | **Positive** | **Negative** | **Null** | **Significant** | **Not Signifi-cant** |
| Dolk et al. [23] (1998); Multi-Europe countries (1982-1994) | Case-control study/Ⅲ | Cases: 45  Controls: 2366 | Prenatal | Distance between the place of residence and the landfill sites | Landfill sites | √ |  |  |  | √ |
| Elliott et al. [24] (2001); Great Britain  (1982-1997) | Case-control study/Ⅲ | Cases: N/A  Controls: N/A | Prenatal | Poisson regression model | Near landfill  (<2 km)  All wastes  Special waste  Non-special waste | √ |  |  | √ |  |
|  |  |  |  |  |  | √ |  |  | √ |  |
|  |  |  |  |  |  | √ |  |  | √ |  |
|  |  |  |  |  |  | √ |  |  | √ |  |
| Morris et al. [25] (2003); Scotland.  (1993-1995) | Case-control study/Ⅲ | Cases: N/A Controls: N/A | Prenatal | Poisson regression model | < 2 km from a special waste site |  | √ |  |  | √ |
| Cordier et al. [26] (2004); France  (1988-1997) | Case-control study/Ⅲ | Cases: communities exposed:78  unexposed: 364  Controls: N/A | Prenatal | Expert assessments to construct a semi-quantitative estimate of the emissions | MSWI emissions |  | √ |  |  | √ |
| Padula et al. [27] (2013); San Joaquin Valley (1997-2006) | Case-control study/Ⅲ | Cases: 67  Controls: 443 | First 2 months of pregnancy | The station-specific daily air quality data were spatially interpolated using inverse distance-squared weighting | CO | √ |  |  |  | √ |
|  |  |  |  |  | NO | √ |  |  |  | √ |
|  |  |  |  |  | NO_2_ | √ |  |  |  | √ |
|  |  |  |  |  | PM_10_ |  | √ |  |  | √ |
|  |  |  |  |  | PM_2.5_ |  | √ |  |  | √ |
|  |  |  |  |  | O_3_ |  | √ |  |  | √ |
| Vinikoor-Imler et al. [28]  (2013); North Carolina  (2003-2005) | Case-control study/Ⅲ | Cases:  978  Controls:  N/A | Weeks 3  to 8 | A hierarchical Bayesian model that combined modeled air pollution estimates from the U.S. Environmental Protection Agency’s (EPA’s) Community Multi-Scale Air Quality (CMAQ) model | PM_2.5_: |  | √ |  |  | √ |
|  |  |  |  |  | O_3_ |  | √ |  |  | √ |
| Schembari et al. [17] (2014); Barcelona, Spain (1994–2006) | Case-control study/Ⅲ | 1994–2006:  Cases:74  Controls:  2423  2000–2006:  Cases:23  Controls:700 | Weeks 3  to 8 | The spatial land use regression (LUR) modeling | NO_2_ | √ |  |  |  | √ |
|  |  |  |  |  | NO_X_ |  | √ |  |  | √ |
|  |  |  |  |  | PM_2.5 absorbance_ |  | √ |  |  | √ |
|  |  |  |  |  | PM_10_ | √ |  |  |  | √ |
|  |  |  |  |  | PM _coarse_ | √ |  |  |  | √ |
|  |  |  |  |  | PM_2.5_ | √ |  |  |  | √ |
| Vinikoor-Imler et al. [18] (2015); Texas  (2002-2006) | Case-control study/Ⅲ | Cases:369  Controls:  711833 | First trimester | A hierarchical Bayesian model that combined modeled air pollution estimates from the U.S. Environmental Protection Agency’s (EPA’s) Community Multi-Scale Air Quality (CMAQ) model | PM_2.5_ | √ |  |  |  | √ |
|  |  |  |  |  | O_3_ | √ |  |  |  | √ |
| Landau et al. [31] (2015); Israel  (2010-2013) | Cohort study/Ⅲ | N/A /1024 | The first trimester  and 3 months prior to conception | The inverse distance weighting method | NO_2_, SO_2_, O_3_, CO, PM_10_ and PM_2.5_ |  |  |  |  |  |
| Vinceti et al. [29] (2016);  Italy  (1998-2006) | Case-control study/Ⅲ | Cases:3  Control:228 | Within the 9 month period before parturition (or 3 months before induced abortion) | Based on the California Line Source Dispersion Model version 4 | PM_10_ |  | √ |  |  | √ |
| Ren et al. [19] (2018); Ohio  (2006-2010) | Cohort study/Ⅱ | **10-km cohort:**  111/ 146114  **7-km cohort:**  78/ 105920  **5-km cohort:**  50/ 72225 | 1 and 2 months before  conception, the month of conception, and the average of 3 months | PM_2.5_ levels were measured by 57 US Environmental Protection Agency stationary monitors across Ohio, and from this monthly averages were calculated | **PM_2.5_ 10-km cohort** |  | | | | |
|  |  |  |  |  | **2 months before** |  |  |  |  |  |
|  |  |  |  |  | per IQR |  |  | √ |  | √ |
|  |  |  |  |  | per 10 um/m^3^ | √ |  |  |  | √ |
|  |  |  |  |  | **1 month before** |  |  |  |  |  |
|  |  |  |  |  | per IQR | √ |  |  |  | √ |
|  |  |  |  |  | per 10 um/m^3^ | √ |  |  |  | √ |
|  |  |  |  |  | **Month of conception** |  |  |  |  |  |
|  |  |  |  |  | per IQR | √ |  |  | √ |  |
|  |  |  |  |  | per 10 um/m^3^ | √ |  |  | √ |  |
|  |  |  |  |  | **Average of 3 months** |  |  |  |  |  |
|  |  |  |  |  | per IQR | √ |  |  |  | √ |
|  |  |  |  |  | per 10 um/m^3^ | √ |  |  |  | √ |
|  |  |  |  |  | **7-km cohort** |  | | | | |
|  |  |  |  |  | **2 months before** |  |  |  |  |  |
|  |  |  |  |  | per IQR | √ |  |  |  | √ |
|  |  |  |  |  | per 10 um/m^3^ | √ |  |  |  | √ |
|  |  |  |  |  | **1 month before** |  |  |  |  |  |
|  |  |  |  |  | per IQR | √ |  |  | √ |  |
|  |  |  |  |  | per 10 um/m^3^ | √ |  |  | √ |  |
|  |  |  |  |  | **Month of conception** |  |  |  |  |  |
|  |  |  |  |  | per IQR | √ |  |  | √ |  |
|  |  |  |  |  | per 10 um/m^3^ | √ |  |  | √ |  |
|  |  |  |  |  | **Average of 3 months** |  |  |  |  |  |
|  |  |  |  |  | per IQR | √ |  |  | √ |  |
|  |  |  |  |  | per 10 um/m^3^ | √ |  |  | √ |  |
|  |  |  |  |  | **5-km cohort** |  | | | | |
|  |  |  |  |  | **2 months before** |  |  |  |  |  |
|  |  |  |  |  | per IQR | √ |  |  |  | √ |
|  |  |  |  |  | per 10 um/m^3^ | √ |  |  |  | √ |
|  |  |  |  |  | **1 month before** |  |  |  |  |  |
|  |  |  |  |  | per IQR | √ |  |  | √ |  |
|  |  |  |  |  | per 10 um/m^3^ | √ |  |  | √ |  |
|  |  |  |  |  | **Month of conception** |  |  |  |  |  |
|  |  |  |  |  | per IQR | √ |  |  |  | √ |
|  |  |  |  |  | per 10 um/m^3^ | √ |  |  |  | √ |
|  |  |  |  |  | **Average of 3 months** |  |  |  |  |  |
|  |  |  |  |  | per IQR | √ |  |  | √ |  |
|  |  |  |  |  | per 10 um/m^3^ | √ |  |  | √ |  |
| Salavati et al. [10] (2018); Netherlands  (1999-2014) | Case-control study/Ⅲ | Cases:446  Controls(1):  775  Cases:446  Controls(2):  2634 | The periconceptional period | Land use regression (LUR) models | **control group 1** |  | | | | |
|  |  |  |  |  | NO_2_ |  |  | √ |  | √ |
|  |  |  |  |  | NO_X_ |  | √ |  |  | √ |
|  |  |  |  |  | PM_10_ |  | √ |  |  | √ |
|  |  |  |  |  | PM_2.5_ | √ |  |  |  | √ |
|  |  |  |  |  | PM_10-2.5_ |  | √ |  |  | √ |
|  |  |  |  |  | **control group 2** |  | | | | |
|  |  |  |  |  | NO_2_ | √ |  |  | √ |  |
|  |  |  |  |  | NO_X_ | √ |  |  | √ |  |
|  |  |  |  |  | PM_10_ | √ |  |  |  | √ |
|  |  |  |  |  | PM_2.5_ | √ |  |  |  | √ |
|  |  |  |  |  | PM_10-2.5_ | √ |  |  | √ |  |
| Sheth et al. [30] (2019); Texas  (1999-2008) | Case-control study/Ⅲ | Cases:8981  Controls:89810 | Prenatal | The 2005 NATA Hazardous Air Pollutant Exposure  Model, version 5 (HAPEM5) | Biphenyl  (high vs low) |  | √ |  |  | √ |
|  |  |  |  |  | 4-Nitrophenol | √ |  |  |  | √ |
|  |  |  |  |  | Bis(2-ethylhexyl)  phthalate |  | √ |  |  | √ |
|  |  |  |  |  | Cresols | √ |  |  |  | √ |
|  |  |  |  |  | Dibutylphthalate | √ |  |  |  | √ |
|  |  |  |  |  | Dimethyl phthalate | √ |  |  | √ |  |
|  |  |  |  |  | Naphthalene |  | √ |  |  | √ |
|  |  |  |  |  | Pentachlorophenol | √ |  |  | √ |  |
|  |  |  |  |  | Phenol | √ |  |  |  | √ |
|  |  |  |  |  | Polychlorinated biphenyls | √ |  |  |  | √ |
| White et al. [21] (2019); Texas, U.S.  (1999-2008) | Case-control study/Ⅲ | Cases:8981  Controls:89810 | Prenatal | The 2005 NATA Hazardous Air  Pollutant Exposure Model, version 5 (HAPEM5) | Arsenic  (high vs low) | √ |  |  | √ |  |
|  |  |  |  |  | Cadmium |  |  | √ |  | √ |
|  |  |  |  |  | Chromium | √ |  |  | √ |  |
|  |  |  |  |  | Lead | √ |  |  | √ |  |
|  |  |  |  |  | Manganese | √ |  |  | √ |  |
|  |  |  |  |  | Mercury | √ |  |  | √ |  |
|  |  |  |  |  | Nickel | √ |  |  |  | √ |
| Parkes et al. [22] (2020);  England and Scotland  (2003-2010) | Cohort study/Ⅱ | 407/ 216004 | Over 91 day pre-pregnancy period plus first trimester of pregnancy | Dispersion model; calculated as a continuous measure of straight line distance of the MWI coordinates to the postcode centroid of mother's residence | PM_10_ |  |  | √ |  | √ |
|  |  |  |  |  | Proximity to nearest MWI | √ |  |  | √ |  |
| Huang et al. [20] (2020);  China  (2007–2014) | Case-control study/Ⅲ | Cases:200  Controls:  2000 | From 3 months before to 6 months after conception | The ordinary kriging method | **PM_10_** |  | | | | |
|  |  |  |  |  | Pre-conception |  |  |  |  |  |
|  |  |  |  |  | 0–1 month | √ |  |  |  | √ |
|  |  |  |  |  | 1–2 month | √ |  |  |  | √ |
|  |  |  |  |  | 2–3 month |  |  | √ |  | √ |
|  |  |  |  |  | 0–3 month | √ |  |  |  | √ |
|  |  |  |  |  | **Post-conception** |  | | | | |
|  |  |  |  |  | 0–1 month | √ |  |  |  | √ |
|  |  |  |  |  | 1–2 month | √ |  |  |  | √ |
|  |  |  |  |  | 2–3 month | √ |  |  |  | √ |
|  |  |  |  |  | 0–3 month: | √ |  |  |  | √ |
|  |  |  |  |  | 3–4 month | √ |  |  |  | √ |
|  |  |  |  |  | 4–5 month | √ |  |  |  | √ |
|  |  |  |  |  | 5–6 month | √ |  |  |  | √ |
|  |  |  |  |  | 3–6 month | √ |  |  |  | √ |
|  |  |  |  |  | **PM_2.5_** |  | | | | |
|  |  |  |  |  | Pre-conception |  |  |  |  |  |
|  |  |  |  |  | 0–1 month | √ |  |  |  | √ |
|  |  |  |  |  | 1–2 month | √ |  |  |  | √ |
|  |  |  |  |  | 2–3 month |  | √ |  |  | √ |
|  |  |  |  |  | 0–3 month | √ |  |  |  | √ |
|  |  |  |  |  | **Post-conception** |  | | | | |
|  |  |  |  |  | 0–1 month | √ |  |  |  | √ |
|  |  |  |  |  | 1–2 month | √ |  |  |  | √ |
|  |  |  |  |  | 2–3 month | √ |  |  | √ |  |
|  |  |  |  |  | 0–3 month: | √ |  |  | √ |  |
|  |  |  |  |  | 3–4 month | √ |  |  |  | √ |
|  |  |  |  |  | 4–5 month | √ |  |  |  | √ |
|  |  |  |  |  | 5–6 month | √ |  |  |  | √ |
|  |  |  |  |  | 3–6 month | √ |  |  |  | √ |
|  |  |  |  |  | **PM_2.5–10_** |  | | | | |
|  |  |  |  |  | Pre-conception |  |  |  |  |  |
|  |  |  |  |  | 0–1 month |  | √ |  |  | √ |
|  |  |  |  |  | 1–2 month |  |  | √ |  | √ |
|  |  |  |  |  | 2–3 month | √ |  |  |  | √ |
|  |  |  |  |  | 0–3 month |  |  | √ |  | √ |
|  |  |  |  |  | **Post-conception** |  | | | | |
|  |  |  |  |  | 0–1 month |  | √ |  |  | √ |
|  |  |  |  |  | 1–2 month | √ |  |  |  | √ |
|  |  |  |  |  | 2–3 month | √ |  |  |  | √ |
|  |  |  |  |  | 0–3 month |  |  | √ |  | √ |
|  |  |  |  |  | 3–4 month |  |  | √ |  | √ |
|  |  |  |  |  | 4–5 month |  | √ |  |  | √ |
|  |  |  |  |  | 5–6 month |  | √ |  |  | √ |
|  |  |  |  |  | 3–6 month |  | √ |  |  | √ |
|  |  |  |  |  | **NO_2_** |  | | | | |
|  |  |  |  |  | Pre-conception |  |  |  |  |  |
|  |  |  |  |  | 0–1 month |  | √ |  |  | √ |
|  |  |  |  |  | 1–2 month |  | √ |  |  | √ |
|  |  |  |  |  | 2–3 month |  | √ |  |  | √ |
|  |  |  |  |  | 0–3 month |  | √ |  |  | √ |
|  |  |  |  |  | **Post-conception** |  | | | | |
|  |  |  |  |  | 0–1 month |  | √ |  |  | √ |
|  |  |  |  |  | 1–2 month | √ |  |  |  | √ |
|  |  |  |  |  | 2–3 month | √ |  |  |  | √ |
|  |  |  |  |  | 0–3 month | √ |  |  |  | √ |
|  |  |  |  |  | 3–4 month | √ |  |  |  | √ |
|  |  |  |  |  | 4–5 month | √ |  |  |  | √ |
|  |  |  |  |  | 5–6 month |  |  | √ |  |  |
|  |  |  |  |  | 3–6 month | √ |  |  |  | √ |
|  |  |  |  |  | **NOx** |  | | | | |
|  |  |  |  |  | Pre-conception |  |  |  |  |  |
|  |  |  |  |  | 0–1 month |  | √ |  |  | √ |
|  |  |  |  |  | 1–2 month |  | √ |  |  | √ |
|  |  |  |  |  | 2–3 month |  | √ |  |  | √ |
|  |  |  |  |  | 0–3 month |  | √ |  |  | √ |
|  |  |  |  |  | **Post-conception** |  | | | | |
|  |  |  |  |  | 0–1 month |  | √ |  |  | √ |
|  |  |  |  |  | 1–2 month | √ |  |  |  | √ |
|  |  |  |  |  | 2–3 month | √ |  |  |  | √ |
|  |  |  |  |  | 0–3 month: |  | √ |  |  | √ |
|  |  |  |  |  | 3–4 month |  | √ |  |  | √ |
|  |  |  |  |  | 4–5 month |  | √ |  |  | √ |
|  |  |  |  |  | 5–6 month |  | √ |  |  | √ |
|  |  |  |  |  | 3–6 month |  | √ |  |  | √ |
|  |  |  |  |  | **O_3_** |  | | | | |
|  |  |  |  |  | Pre-conception |  |  |  |  |  |
|  |  |  |  |  | 0–1 month |  | √ |  |  | √ |
|  |  |  |  |  | 1–2 month |  | √ |  |  | √ |
|  |  |  |  |  | 2–3 month |  | √ |  |  | √ |
|  |  |  |  |  | 0–3 month |  | √ |  |  | √ |
|  |  |  |  |  | **Post-conception** |  | | | | |
|  |  |  |  |  | 0–1 month | √ |  |  | √ |  |
|  |  |  |  |  | 1–2 month | √ |  |  |  | √ |
|  |  |  |  |  | 2–3 month | √ |  |  |  | √ |
|  |  |  |  |  | 0–3 month | √ |  |  |  | √ |
|  |  |  |  |  | 3–4 month |  | √ |  |  | √ |
|  |  |  |  |  | 4–5 month | √ |  |  |  | √ |
|  |  |  |  |  | 5–6 month | √ |  |  |  | √ |
|  |  |  |  |  | 3–6 month | √ |  |  |  | √ |
|  |  |  |  |  | **O_3_ 8-h max** |  | | | | |
|  |  |  |  |  | Pre-conception |  |  |  |  |  |
|  |  |  |  |  | 0–1 month |  | √ |  |  | √ |
|  |  |  |  |  | 1–2 month |  | √ |  |  | √ |
|  |  |  |  |  | 2–3 month |  | √ |  |  | √ |
|  |  |  |  |  | 0–3 month |  | √ |  |  | √ |
|  |  |  |  |  | **Post-conception** |  | | | | |
|  |  |  |  |  | 0–1 month | √ |  |  |  | √ |
|  |  |  |  |  | 1–2 month | √ |  |  |  | √ |
|  |  |  |  |  | 2–3 month | √ |  |  |  | √ |
|  |  |  |  |  | 0–3 month | √ |  |  |  | √ |
|  |  |  |  |  | 3–4 month |  | √ |  |  | √ |
|  |  |  |  |  | 4–5 month | √ |  |  |  | √ |
|  |  |  |  |  | 5–6 month | √ |  |  |  | √ |
|  |  |  |  |  | 3–6 month | √ |  |  |  | √ |

Abbreviations: CO, carbon monoxide; IQR, interquartile range; MWI, municipal waste incinerators; N/A, not available; NO, nitric oxide; NO_2_, nitrogen oxide; NO_X_, nitrogen oxides; O_3_, ozone; PM _coarse_, particulate matter coarse; PM_10_, particulate matter with aerodynamic diameter ≤10 μm; PM_10-2.5_, the coarse fraction of particulate matter; PM_2.5_, particulate matter with aerodynamic diameter ≤ 2.5 μm; PM_2.5 absorbance_, particulate matter with aerodynamic diameter ≤ 2.5 μm absorbance; PM_2.5-10_, particulate matter with aerodynamic diameter 2.5-10 μm.
