# Supplementary material for: Association between prenatal air pollution exposure and risk of hypospadias in offspring: a systematic review and meta-analysis of observational studies
Source: Aging (Albany NY). 2021 Mar 19;13(6):8865–79. doi: 10.18632/aging.202698 (PMC8034939; doi:10.18632/aging.202698)
Supplement: Supplementary Table 2 [file aging-13-202698-s002.docx]

**Supplementary Table 2. Detail findings of studies included in the meta-analysis for association between prenatal air pollution and risk of hypospadias.**

| **Author’s name [Ref], year** | **Exposure Variable** | **Main findings related to hypospadias defects**  **OR (95% CI)** |
| --- | --- | --- |
| Dolk et al.  [23], 1998 | Categorical | within 3 km:1.96 (0.98-3.92) |
| Elliott et al. [24], 2001 | Categorical | Near landfill (<2 km):1.07 (1.04-1.10)  All wastes:1.07 (1.04-1.10)  Special waste:1.11 (1.03-1.21)  Non-special waste: 1.07 (1.04-1.11) |
| Morris et al. [25], 2003 | Categorical | < 2 km from a special waste sit: 0.84 (0.58-1.22) |
| Cordier et al. [26], 2004 | Categorical | 0.88 (0.66-1.19) |
| Padula et al. [27], 2013 | Categorical | CO: 1.4 (0.6-3.0)  NO: 1.7 (0.8-3.7)  NO_2_: 1.1 (0.5-2.4)  PM_10_: 0.8 (0.4-1.8)  PM_2.5_: 0.8 (0.3-2.0)  O_3_ 8–hour maximum: 0.7 (0.3-1.5) |
| Vinikoor-Imler et al. [28], 2013 | Continuous | PM_2.5_: 0.98 (0.90-1.07)  O_3_: 0.95 (0.86-1.06) |
| Schembari et al. [17], 2014 | Continuous | 1994–2006:  NO_2_: 1.02 (0.78-1.34)  NO_X_: 0.98 (0.91-1.05)  PM_2.5 absorbance_ :0.89 (0.57-1.39)  2000–2006:  PM_10_: 1.15 (0.94-1.41)  PM _coarse_: 1.15 (0.77-1.70)  PM_2.5_: 1.18 (0.87-1.60) |
| Vinikoor-Imler et al. [18], 2015 | Continuous | PM_2.5_: 1.14 (0.89-1.44)  O_3_: 1.20 (0.92-1.57) |
| Landau et al.  [31], 2015 | N/A | N/A |
| Vinceti et al. [29], 2016 | Continuous | PM_10_: OR^2^ 0.56 (0.11-2.88) |
| Ren et al.  [19], 2018 | Continuous | 10 km cohort:  2 months before  per IQR increment:1.00 (0.77-1.31)  per 10 um/m^3^ increment: 1.01 (0.59-1.72)  1 month before  per IQR increment: 1.16 (0.96-1.40)  per 10 um/m^3^ increment: 1.36 (0.92-2.00)  Month of conception  per IQR increment: 1.39 (1.07-1.81)  per 10 um/m^3^ increment: 1.97 (1.14-3.38)  Average of 3 months  per IQR increment: 1.27 (0.99-1.61)  per 10 um/m^3^ increment: 1.97 (0.98-3.96)  7 km cohort:  2 months before  per IQR increment: 1.02 (0.79-1.31)  per 10 um/m^3^ increment: 1.03 (0.62-1.73)  1 month before  per IQR increment: 1.39 (1.15-1.69)  per 10 um/m^3^ increment: 2.00 (1.34-2.95)  Month of conception  per IQR increment: 1.47 (1.06-2.02)  per 10 um/m^3^ increment: 2.18 (1.13-4.20)  Average of 3 months  per IQR increment: 1.44 (1.07-1.94)  per 10 um/m^3^ increment: 2.82 (1.21-6.58)  5 km cohort  2 months before  per IQR increment: 1.22 (0.93-1.59)  per 10 um/m^3^ increment: 1.48 (0.87-2.54)  1 month before  per IQR increment: 1.41 (1.08-1.85)  per 10 um/m^3^ increment: 2.05 (1.17-3.59)  Month of conception  per IQR increment: 1.39 (0.96-2.02)  per 10 um/m^3^ increment: 1.96 (0.92-4.17)  Average of 3 months  per IQR increment: 1.53 (1.10-2.14)  per 10 um/m^3^ increment: 3.35 (1.31-8.61) |
| Salavati et al. [10], 2018 | Continuous | With control group 1  NO_2_: 1.00 (0.97-1.04)  NO_X_: 0.99 (0.97-1.02)  PM_10_: 0.99 (0.80-1.21)  PM_2.5_: 1.24 (0.83-1.86)  PM_10-2.5_: 0.96 (0.66-1.41)  With control group 2  NO_2_: 1.04 (1.01-1.07)  NO_X_: 1.02 (1.00-1.04)  PM_10_: 1.15 (0.97-1.37)  PM_2.5_: 1.29 (0.94-1.78)  PM_10-2.5_: 1.46 (1.05-2.03) |
| Sheth et al. [30], 2019 | Categorical | Biphenyl:  Medium–low: 1.08 (1.01-1.16)  Medium: 1.08 (1.01-1.16)  Medium–high: 1.06 (0.99-1.14)  High: 0.95 (0.88-1.02)  4-Nitrophenol:  Medium–low: 1.04 (0.97-1.12)  Medium: 1.04 (0.97-1.11)  Medium–high: 1.07 (0.99-1.15)  High: 1.04 (0.96-1.12)  Bis(2-ethylhexyl)phthalate:  Medium–low: 0.90 (0.82-0.99)  Medium: 0.97 (0.88-1.06)  Medium–high: 0.92 (0.83-1.01)  High: 0.95 (0.87-1.05)  Cresols:  Medium–low: 1.02 (0.95-1.10)  Medium: 0.97 (0.90-1.06)  Medium–high: 1.06 (0.98-1.14)  High: 1.01 (0.92-1.10)  Dibutylphthalate:  Medium–low: 1.07 (0.99-1.14)  Medium: 1.07 (1.00-1.15)  Medium–high: 1.09 (1.01-1.17)  High: 1.03 (0.96-1.11)  Dimethyl phthalate  Medium–low: 1.01 (0.94-1.09)  Medium: 1.07 (1.00-1.15)  Medium–high: 1.09 (1.01-1.17)  High: 1.15 (1.07-1.23)  Naphthalene  Medium–low: 1.04 (0.96-1.12)  Medium: 1.06 (0.98-1.15)  Medium–high: 1.03 (0.95-1.12)  High: 0.99 (0.90-1.09)  Pentachlorophenol  Medium–low: 1.04 (0.96-1.14)  Medium: 0.99 (0.90-1.09)  Medium–high: 1.06 (0.98-1.15)  High: 1.10 (1.01-1.20)  Phenol  Medium–low: 1.19 (1.11-1.28)  Medium: 1.27 (1.19-1.37)  Medium–high: 1.16 (1.08-1.24)  High: 1.04 (0.97-1.12)  Polychlorinated biphenyls  Medium–low: 1.07 (0.99-1.15)  Medium: 1.11 (1.03-1.20)  Medium–high: 1.08 (1.00-1.16)  High: 1.03 (0.95-1.11) |
| White et al. [21], 2019 | Categorical | Arsenic:  Medium-low: 1.00 (0.93-1.07)  Medium: 1.08 (1.01-1.16)  Medium-high: 1.01 (0.94-1.09)  High: 1.18 (1.10-1.27)  Cadmium:  Medium-low: 1.04 (0.96-1.11)  Medium: 1.13 (1.05-1.21)  Medium-high: 1.11 (1.03-1.19)  High: 1.00 (0.93-1.08)  Chromium:  Medium-low: 1.08 (1.00-1.16)  Medium: 1.05 (0.97-1.12)  Medium-high: 1.12 (1.04-1.20)  High: 1.10 (1.02-1.19)  Lead:  Medium-low: 0.99 (0.92-,1.07)  Medium: 1.04 (0.97-1.12)  Medium-high: 1.13 (1.05-1.21)  High: 1.20 (1.11-,1.28)  Manganese:  Medium-low: 1.06 (0.98-1.14)  Medium: 1.12 (1.04-1.20)  Medium-high: 1.18 (1.10-1.27)  High: 1.11 (1.03-1.20)  Mercury:  Medium-low: 1.06 (0.99-1.14)  Medium: 1.16 (1.08-1.24)  Medium-high: 1.14 (1.06-1.23)  High: 1.08 (1.00-1.16)  Nickel:  Medium-low: 1.03 (0.95-1.10)  Medium: 1.14 (1.06-1.22)  Medium-high: 1.09 (1.02-1.17)  High: 1.04 (0.97-1.12) |
| Parkes et al. [22], 2020 | Continuous | PM_10_: 1.00 (0.90-1.12)  Proximity to nearest MWI: 1.07 (1.01-1.12) |
| Huang et al. [20], 2020 | Continuous | PM_10_  Pre-conception  0–1 month:1.03 (0.79-1.34)  1–2 month:1.02 (0.80-1.31)  2–3 month:1.00 (0.78-1.28)  0–3 month:1.02 (0.81-1.29)  Post-conception  0–1 month: 1.07 (0.83-1.39)  1–2 month: 1.14 (0.90-1.46)  2–3 month: 1.21 (0.95-1.55)  0–3 month: 1.15 (0.91-1.45)  3–4 month: 1.11 (0.86-1.44)  4–5 month: 1.06 (0.83-1.35)  5–6 month: 1.08 (0.84-1.39)  3–6 month: 1.09 (0.86-1.39)  PM_2.5_  Pre-conception  0–1 month: 1.11 (0.85-1.46)  1–2 month: 1.05 (0.81-1.36)  2–3 month: 0.97 (0.75-1.26)  0–3 month: 1.05 (0.81-1.35)  Post-conception  0–1 month: 1.23 (0.95-1.60)  1–2 month: 1.23 (0.96-1.58)  2–3 month: 1.32 (1.02-1.71)  0–3 month: 1.29 (1.01-1.65)  3–4 month: 1.21 (0.92-1.57)  4–5 month: 1.15 (0.89-1.48)  5–6 month: 1.18 (0.90-1.54)  3–6 month: 1.20 (0.93-1.54)  PM_2.5–10_  Pre-conception  0–1 month: 0.95 (0.77-1.18)  1–2 month: 1.00 (0.81-1.23)  2–3 month: 1.03 (0.83-1.27)  0–3 month: 1.00 (0.82-1.21)  Post-conception  0–1 month: 0.91 (0.73-1.14)  1–2 month: 1.02 (0.83-1.26)  2–3 month: 1.08 (0.88-1.31)  0–3 month: 1.00 (0.83-1.22)  3–4 month: 1.00 (0.81-1.24)  4–5 month: 0.96 (0.78-1.18)  5–6 month: 0.99 (0.81-1.21)  3–6 month: 0.98 (0.80-1.20)  NO_2_  Pre-conception  0–1 month: 0.97 (0.69-1.36)  1–2 month: 0.76 (0.55-1.07)  2–3 month: 0.89 (0.65-1.21)  0–3 month: 0.85 (0.59-1.21)  Post-conception  0–1 month: 0.94 (0.68-1.30)  1–2 month: 1.13 (0.80-1.59)  2–3 month: 1.14 (0.82-1.59)  0–3 month: 1.07 (0.75-1.52)  3–4 month: 1.01 (0.73-1.41)  4–5 month: 1.03 (0.74-1.44)  5–6 month: 1.00 (0.71-1.42)  3–6 month: 1.02 (0.71-1.46)  NO_x_  Pre-conception  0–1 month: 0.99 (0.70-1.39)  1–2 month: 0.75 (0.54-1.05)  2–3 month: 0.87 (0.63-1.18)  0–3 month: 0.84 (0.59-1.20)  Post-conception  0–1 month: 0.88 (0.64-1.21)  1–2 month: 1.08 (0.77-1.51)  2–3 month: 1.05 (0.76-1.45)  0–3 month: 0.99 (0.69-1.40)  3–4 month: 0.95 (0.68-1.33)  4–5 month: 0.91 (0.66-1.26)  5–6 month: 0.92 (0.66-1.29)  3–6 month: 0.92 (0.65-1.30)  O_3_  Pre-conception  0–1 month: 0.95 (0.73-1.25)  1–2 month: 0.97 (0.77-1.22)  2–3 month: 0.89 (0.70-1.13)  0–3 month: 0.92 (0.73-1.16)  Post-conception  0–1 month: 1.40(1.08-1.82)  1–2 month: 1.02 (0.81-1.28)  2–3 month: 1.11 (0.84-1.47)  0–3 month: 1.18 (0.95-1.47)  3–4 month: 0.91 (0.67-1.23)  4–5 month: 1.08 (0.86-1.36)  5–6 month: 1.04 (0.80-1.36)  3–6 month: 1.02 (0.80-1.30)  O_3_ 8-hour maximum  Pre-conception  0–1 month: 0.98 (0.78-1.24)  1–2 month: 0.92 (0.74-1.15)  2–3 month: 0.91 (0.74-1.11)  0–3 month: 0.92 (0.74-1.14)  Post-conception  0–1 month: 1.25 (0.99-1.58)  1–2 month: 1.09 (0.88-1.34)  2–3 month: 1.11 (0.87-1.42)  0–3 month: 1.17 (0.95-1.45)  3–4 month: 0.87 (0.67-1.12)  4–5 month: 1.04 (0.84-1.29)  5–6 month: 1.11 (0.89-1.39)  3–6 month: 1.01 (0.81-1.26) |

Abbreviations: CI, confidence interval; CO, carbon monoxide; IQR, interquartile range; MWI, municipal waste incinerators; N/A, not available; NO, nitric oxide; NO_2_, nitrogen oxide; NO_X_, nitrogen oxides; O_3_, ozone; OR, odds ratio; PM _coarse_, particulate matter coarse; PM_10_, particulate matter with aerodynamic diameter ≤10 μm; PM_10-2.5_, the coarse fraction of particulate matter; PM_2.5_, particulate matter with aerodynamic diameter ≤ 2.5 μm; PM_2.5 absorbance_, particulate matter with aerodynamic diameter ≤ 2.5 μm absorbance; PM_2.5-10_, particulate matter with aerodynamic diameter 2.5-10 μm.
