# Supplementary material for: Association between prenatal air pollution exposure and risk of hypospadias in offspring: a systematic review and meta-analysis of observational studies
Source: Aging (Albany NY). 2021 Mar 19;13(6):8865–79. doi: 10.18632/aging.202698 (PMC8034939; doi:10.18632/aging.202698)
Supplement: Supplementary Table 3 [file aging-13-202698-s003.doc]

**Supplementary Table 3. Criteria for the risk of bias assessment of each study, adapted from the OHAT.**

| **Bias categories and questions** | **Definitely Low Risk (“++”)** | **Probably Low Risk (“+”)** | **Probably High Risk**  **(“-” or “NR”: not reported)** | **Definitely High Risk (“--")** |
| --- | --- | --- | --- | --- |
| **Selection Bias (SB)**  comparison group | The descriptions of the studied population are sufficiently detailed to support the assertion that risk of selection effects is minimal. | There is insufficient information about population selection to permit a judgment of low risk of bias, but there is indirect evidence that suggests low risk of bias. | There is insufficient information about population selection to permit a judgment of high risk of bias, but there is indirect evidence that suggests high risk of bias. | There are indications from descriptions of the studied population of high risk of bias. |
| **Confounding Bias (CB)** design and analysis | Study accounts for all important confounders which are measured consistently. | Study accounts for most of confounders AND is not expected to introduce bias. | Study accounts for some but not all of confounders AND is expected to introduce bias. | Study do not account for potential confounders OR are inappropriately measured. |
| **Confounding Bias (CB)** variables assessment | There is direct evidence that primary covariates and confounders are assessed using valid and reliable measurements. | There is evidence (direct or indirect) that primary covariates and confounders are assessed using valid and reliable measurements, OR the measures used would not produce appreciable bias. | There is indirect evidence that primary covariates and confounders are assessed using measurements of unknown validity. | There is direct evidence that primary covariates and confounders are assessed using non valid measurements. |
| **Confounding Bias (CB)** other exposures | There is direct evidence that other exposures anticipated to bias results are not present or are appropriately measured and adjusted for. | There is evidence (direct or indirect) that other co-exposures anticipated to bias results are not present or are appropriately adjusted for, OR co-exposures present would not produce appreciable bias. | There is indirect evidence that an unbalanced provision of additional co-exposures is involved in the primary study groups, which is not appropriately adjusted for. | There is direct evidence that an unbalanced provision of additional co-exposures is involved in the primary study groups, which is not appropriately adjusted for. |
| **Performance Bias (PB)** adhere to protocol | There is direct evidence that no protocol deviation involves in the study. | There is indirect evidence that no protocol deviation involves in the study. | There is indirect evidence that large protocol deviation involves in the study. | There is direct evidence that large protocol deviation involves in the study. |
| **Attrition/Exclusion Bias (A/EB)** outcome data complete | There is no missing outcome data or missing data unrelated to true outcome. | There is insufficient information about incomplete data to judge for low risk, but indirect evidence that suggests low risk of bias. | There is insufficient information about incomplete data to judge for high risk, but indirect evidence that suggests high risk. | Missing outcome data is related to true outcome. |
| **Detection Bias (DB)**  outcomes blinded | Outcome assessors are adequately blinded to the exposure level when outcome is assessed. | There is indirect evidence that the outcome assessors are adequately blinded when reporting outcomes, OR lack of adequate blinding of outcome assessors would not produce appreciable bias. | There is insufficient information provided about blinding of outcome assessors. | Lack of adequate blinding of outcome assessors, including no blinding or incomplete blinding. |
| **Detection Bias (DB)**  exposure characterization    List of major considerations: 1) air pollution measurements were performed daily, < 25% missing data 2) more than one monitoring station per a large geographical area  3) models were used for weighting | There is high confidence that the exposure to PM is the true average population exposure. | There is indirect evidence that suggests low risk of bias, or one of the three listed considerations is not applied. | There is insufficient information to permit a judgment of high risk of bias, but there is indirect evidence that suggests high risk of bias. Additionally, two out of the three listed considerations are not applied. | There is direct evidence of high risk of misclassification bias, or all three of the listed considerations are not applied. |
| **Detection Bias (DB)**  outcome assessment | Outcome is classified based on diagnosis standard criteria (International Classification System code) and provided by a national or regional database. | Outcome is assessed based on diagnosis standard criteria and collected by researcher. | Outcome is not assessed based on standard diagnosis criteria AND is accompanied by validation sub-study or sensitivity analysis to suggest that the risk is minimum. | Outcome is assessed based on self-reports (parents, family) and data collected by the researcher. |
| **Selective Reporting Bias (SRB)**  outcomes reported | All of the studies pre-specified outcomes and findings are reported. | There is insufficient information about selective outcome to judge for low risk, but indirect evidence that suggests study is free of selective report. | There is insufficient information about selective reporting to judge for high risk, but indirect evidence suggests that study is not free of selective report. | Not all pre-specified outcomes and findings are reported, or one/more of the primary outcomes or analyses is/are assessed or executed with other methods than the pre-specified one, or one/more of the reported outcomes/findings is/are not pre-specified. |
| **Other Bias (OB)** | No other sources of bias. | There is insufficient information to judge for low risk, but indirect evidence suggests study is free of other problems. | There is insufficient information to judge for high risk, but indirect evidence suggests study is not free of other problems. | There is at least one important risk of bias. |
